# Effects of Auricular Point Acupressure with Lifestyle Interventions on Cerebrovascular Function among Adults with High Stroke Risk: A Randomized Controlled Study

**DOI:** 10.1155/2023/6879150

**Published:** 2023-04-19

**Authors:** Le Luo, Liu Huang, Shi-Jie Han, Du Wu, Yue Qian, Ke-Qin Jiang, Lei Yang

**Affiliations:** ^1^WuYunShan Hospital of Hangzhou, Hangzhou, Zhejiang 311121, China; ^2^Department of Health Management, Faculty of Public Health, School of Medicine, Hangzhou Normal University, Hangzhou, Zhejiang 311121, China

## Abstract

**Background:**

To investigate the potential benefits of the auricular point acupressure on cerebrovascular function and stroke prevention among adults with high stroke risk.

**Methods:**

A randomized controlled study was performed in 105 adults at high risk for stroke between March and July 2021. Participants were randomly allocated (1 : 1) to receive either auricular point acupressure with basic lifestyle interventions (*n* = 53) or basic lifestyle interventions alone (*n* = 52) for 2 weeks. The primary outcome was the kinematic and dynamic indices of cerebrovascular function, as well as CVHP score at week 2, measured by the Doppler ultrasonography and pressure transducer on carotids.

**Results:**

Among the 105 randomized subjects, 86 finished the intervention plans. At week 2, the auricular point acupressure therapy with lifestyle intervention group had higher kinematic indices, cerebrovascular hemodynamic parameters score and lower dynamic indices than the lifestyle intervention group.

**Conclusions:**

Cerebrovascular function and cerebrovascular hemodynamic parameters score were greater improved among the participants undergoing auricular point acupressure combined with lifestyle interventions than lifestyle interventions alone. Hence, the auricular point acupressure can assist the stroke prevention. *Trail Registration.* This trial registered with ChiCTR2100041769 on https://www.chictr.org.cn/.

## 1. Introduction

With the aggravation of population ageing, sustaining high prevalence of risk factors of stroke [1], and improper quality management [2], the burden of stroke in China has risen steeply [3] during the past 3 decades. At present, stroke is the prime cause of death in China, results in more than 2 million new cases annually, and brings about the severest burden of stroke [4]. Hence, the prevention of stroke becomes urgent in China, and identifying individuals with a risk for stroke is crucial for controlling the burden of stroke.

Although a number of tools have been established for the assessment of stroke risk, almost all of these risk prediction algorithms, such as Framingham Stroke Risk Profile [5] and Pooled Cohort Equations in the United States, the Systematic Coronary Risk Evaluation model in Europe [6], and the QRISK score in the United Kingdom [7], originated from Western population-based studies. Given that there is largely racial and geographical diversity between Western and Chinese population, these approaches to predicting stroke risk become unreliable for populations in China. In order to tackle the problem, cerebrovascular hemodynamic parameters (CVHPs), aiming at assessing cerebrovascular function, have been published for the evaluation of the stroke risk in China [8].

The CVHP, which contains kinematic and dynamic indices of cerebrovascular function, is collected via Doppler ultrasonography and pressure transducer on carotids. The kinematic indices contain the numerical value of maximal, minimal, and mean velocity of carotid blood flow (*V*_max_, *V*_min_, and *V*_mean_), as well as mean quantity of carotid blood flow (*Q*_mean_). The dynamic indices include the numerical value of peripheral resistance of vessels (Rv), dynamic resistance (DR), pulse wave velocity (WV), characteristic impendence of vessels (Zcv), capillary pressure (CP), and differential pressure (DP) [8]. The CVHP score is calculated according to the weight of each index, and the weight is evaluated by the contribution of the indices to stroke [8]. The score ranges from 0 to 100, and the cut-off value of the CVHP score for stroke risk screening is 75 [9], which means individuals whose CVHP scores are less than 75 are considered to be at high risk for stroke [8]. In addition, findings from a large cohort study with 27184 cases in China have demonstrated that the CVHP score is negatively related with the modified Framingham Stroke Risk Profile score and could be used as a tool for primary prevention of cerebral stroke in China [10].

Stroke is largely associated with unhealthy lifestyle behaviors including nutrition, smoking, inadequate physical activity [11], and hypertension [11]. Though the long-term effect of lifestyle interventions on stroke incidence has not been assessed in randomized trials, accumulating evidence has suggested an inverse association between favorable lifestyle behaviors and stroke risk [12–14]. In addition, some studies have revealed that the auricular point acupressure (APA) therapy may lower the blood pressure of the patients with stroke and hypertension [15]. Nevertheless, the effect of the auricular point acupressure therapy on CVHP is currently unknown. Thus, we conducted a randomized controlled study to investigate the effect of the auricular point acupressure and lifestyle interventions on CVHP and the risk of stroke.

## 2. Methods

### 2.1. Study Design

We performed a single-center, randomized, parallel-group, lifestyle interventions-controlled clinical study at WuYunShan Hospital of Hangzhou, Zhejiang province, China. Informed consent was obtained from all participants recruited in the study, and the study protocols were approved by the Medical Ethical Committee of WuYunShan Hospital of Hangzhou, with the clinical trial registration number ChiCTR2100041769 listed at https://www.chictr.org.cn/. All procedures were in accordance with the Declaration of Helsinki.

### 2.2. Setting and Participants

Subjects with high risk for stroke were recruited from WuYunShan Hospital of Hangzhou, when they attended a routine health examination during March and July 2021. Subjects were eligible if they were 35 to 85 years old, and their CVHP scores were less than 75. Major exclusion criteria were the diagnosis of stroke, malignant tumor, and severe cognitive disorder. Subjects recruited were not compensated for study participation.

### 2.3. Randomization and Blinding

Eligible participants were randomly allocated to an APA therapy with lifestyle intervention group and a lifestyle intervention alone group used as controls, with a 1 : 1 ratio and matching age and gender via the MinimPy [16], which is a randomization program for clinical study.

Whereas participants and healthcare providers allocated to the intervention group were aware of the allocated arm, outcome adjudicators were kept blinded to the allocation by mixing participants and nonparticipants together during the routine assessment process.

### 2.4. Interventions

Participants in the APA therapy with lifestyle intervention group received two therapies, namely the auricular point acupressure therapy and basic lifestyle interventions.

The APA therapy was performed using the Cowherb Seed stickers (Shanghai Taichen Tech&Development Co., Ltd, China) by well-trained acupuncturists. The Cowherb Seed stickers were fixed, by an adhesive backing, onto ten auricular points, which are the bilateral Zhen (AT_3_, located at the 3rd area of Antitragus), Benmen (CO_3_, located at the 3rd area of the concha), Shierzhichang (CO_5_, located at the 5th area of the concha), Shenmen (TF_4_, located at the bifurcation of the crura of antihelix), and Shen (CO_10_, located at the 10th area of the concha). Afterwards, the stickers were renewed every 3 days by the acupuncturists in case of shedding, and let the ear points rest at least eight hours between the sticker placements. The participants were taught to simultaneously press the bilateral auricular points with the seeds on the stickers 3 times daily for 5 minutes each time for 2 weeks, under the guidance of medical staff. Therefore, participants received 3 treatment sessions per day for 2 weeks (14 consecutive days), 42 sessions in total.

Besides, the basic lifestyle intervention strategies mainly included a guidance on dietary and physical activity, which was supported by a wristband fitness tracker with a mobile app named Xuebei Classroom (Hangzhou Xuebei Technology Co., Ltd, China). The fitness tracker helped to measure the activity and send the information to the app, and the app included video and text on lifestyle education, and helped to remind the participants to follow the guidance on lifestyle behaviors.

The participants in the lifestyle intervention group received only the 2-week basic lifestyle interventions, which were identical to those performed in the APA therapy with lifestyle intervention group.

### 2.5. Outcomes

The primary outcomes were the indices of CVHP and CVHP score at week 2 in APA therapy with lifestyle intervention group and the lifestyle intervention group. Well-trained medical staff used GT-3000 (Shanghai Shenzhou Gaote Medical Equipment Co., Ltd, Shanghai, China) hemodynamic analyzer to detect CVHP by Doppler and pressure transducer on both left and right common carotid arteries, and the GT-3000 also calculated the CVHP score for assessment of stroke risk.

### 2.6. Statistical Analysis

In the light of our data of a previous pilot study, a between-group difference of the *V*_mean_ was 1.07 cm/s, and the standard deviation was 1.27 cm/s. A sample size of 31 subjects in each group was estimated to provide 90% power, with the use of a two-sided test at a type I error rate of 5%. In addition, given a 20% loss to the follow-up and discontinued intervention, the sample size of this study finally added up to 38.

The primary outcome was analyzed in accordance with the complete-case analysis principle. The CVHP and CVHP scores at week 2 between the APA therapy with lifestyle intervention group and the lifestyle intervention group were evaluated by using analysis of covariance (ANCOVA) with postmeasures at week 2 as the dependent variable and baseline measures as the covariate. All statistical analyses were performed in SPSS 24.0 software (SPSS Inc., Chicago, IL, USA). The Benjamini–Hochberg procedure with a false discovery rate (FDR) at 0.05 was used to adjust for multiple comparisons in this study [17].

## 3. Results

### 3.1. Participants and Study Treatment

**Figure 1:**
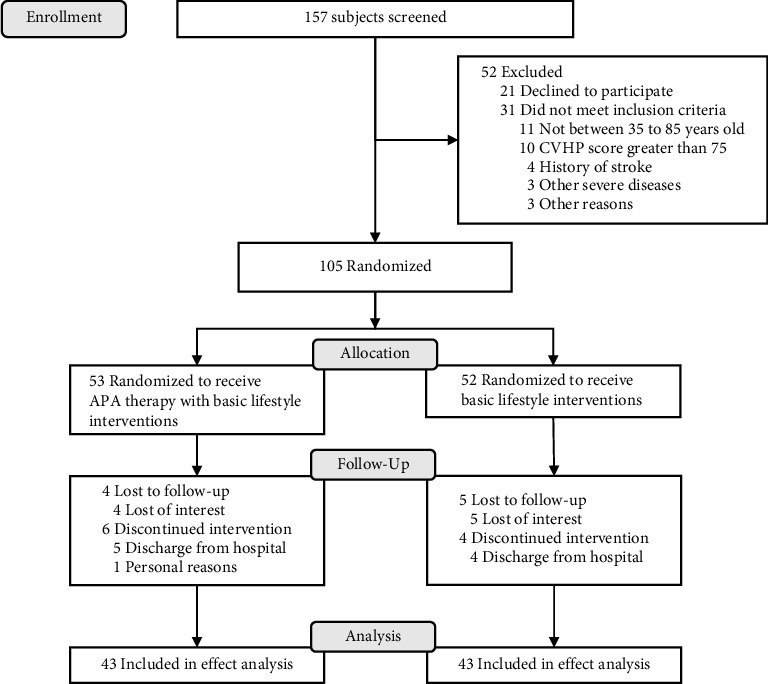
Flowchart of the study design.

During March and July 2021, we screened 157 subjects for eligibility, of whom 21 declined to participate in the study and 31 did not meet the inclusion criteria. The remaining enrollees were randomly assigned to receive either an APA therapy with basic lifestyle interventions (*n* = 53) or basic lifestyle interventions alone (*n* = 52). Among the randomized enrollees, 86 (81.9%, 43 in each group) completed 2 weeks of study intervention (Figure 1). The two groups were similar with respect to all baseline characteristics (Table 1).

### 3.2. Comparisons of the Kinematic Indices of CVHP between the Two Groups

The kinematic indices of CVHP between the APA therapy with lifestyle intervention group and lifestyle intervention group are shown in Table 2. After the 2-week intervention, the right *Q*_mean_ in the two groups was 8.34 ± 1.20 ml/s and 7.78 ± 1.07 ml/s, respectively. Similarly, the left *V*_mean_ in the two groups was 17.30 ± 2.53 cm/s and 15.32 ± 2.23 cm/s, respectively. The right *V*_mean_ in the two groups was 15.98 ± 2.65 cm/s and 13.79 ± 2.20 cm/s, respectively. The right *Q*_mean_, bilateral *V*_mean_, left *V*_max_, and bilateral *V*_min_ in the APA therapy with lifestyle intervention group were higher than those in the lifestyle intervention group (Table 2).

### 3.3. Comparisons of the Dynamic Indices of CVHP between the Two Groups

Table 3 indicates that after the 2-week intervention, the left WV in the APA therapy with lifestyle intervention group and lifestyle intervention group was 15.76 ± 5.25 m/s and 18.54 ± 5.12 m/s, respectively. Likewise, the left Zcv in the two groups were 16.55 ± 5.51 kPa·km/s and 19.47 ± 5.37 kPa·km/s. The left WV, left Zcv, bilateral Rv, and bilateral DR in the APA therapy with lifestyle intervention group were much lower than those in the lifestyle intervention group (Table 3).

### 3.4. Comparisons of the CVHP Score between the Two Groups

Table 4 shows that after the 2-week intervention, CVHP score of the APA therapy with lifestyle intervention group was 85.88 ± 8.89 and CVHP score of the lifestyle intervention group was 69.41 ± 13.39. The CVHP score in the APA therapy with lifestyle intervention group was much higher than that in the lifestyle intervention group (Table 4).

## 4. Discussion

China saw an ongoing increase in the incident and burden of stroke which is the leading cause of death in China [3]. Hence, investigating approaches to the prevention of stroke become a great challenge for medical and health researchers [18–20]. Though evidences have demonstrated that the auricular point acupressure therapy was able to lower blood pressure [15, 21, 22], and the auricular acupuncture and electrical stimulation elevated cortical regional cerebral blood flow in rats model [23, 24], whether the APA can ameliorate the cerebrovascular hemodynamic function of individuals with high risk for stroke was still unknown.

In this randomized controlled study, the cerebrovascular hemodynamic function was assessed by the CVHP which reflects the status of blood flow and blood vessels, measured by the kinematic and dynamic indices respectively. Our findings showed that the kinematic indices, including the *Q*_mean_, *V*_mean_, *V*_max_, and *V*_min_, of participants who received the APA therapy in combination with lifestyle interventions were higher, while the dynamic indices including the WV, Zcv, Rv, and DR were lower, compared with those who received the lifestyle interventions alone. As the kinematic indices mainly reflect the condition of cerebral blood supply, a higher kinematic index value means greater cerebral blood supply, which is of great importance to rescuing salvageable ischemic brain tissue and stroke prevention [25]. In contrast, the dynamic indices mainly present the condition of the cerebrovascular elasticity or stiffness, and a decreased dynamic index value means higher arterial elasticity or lower stiffness, and accumulating evidence has suggested the association between arterial stiffness and stroke attack [26–28]. In addition, the CVHP score of the participants in the APA with lifestyle intervention group was higher than that of the lifestyle intervention alone group. Our findings were consistent with a previous observational study, which has suggested that the value of the CVHP score is negatively associated with the risk of stroke [29]. Therefore, findings of the primary outcomes in our study indicated that over a 2-week intervention, the APA therapy in combination with lifestyle interventions may facilitate the cerebrovascular hemodynamic function and ameliorate the stroke risk among the adults with high risk for stroke.

The physiological mechanism underlying the effect of APA therapy on cerebrovascular hemodynamic function is not fully understood at present. There may be two possible explanations. First, a previous study has demonstrated that auricular acupuncture can directly increase mean blood flow velocity in middle cerebral artery [30]. Second, cerebrovascular hemodynamic function is indirectly influenced by the effect of APA therapy on the blood pressure. It was well known that the APA therapy can lower the blood pressure [15, 21, 22], while the cerebral blood flow was believed to be autoregulated and near constant over a wide blood pressure range and therefore was not largely affected by the change of the blood pressure. However, a very recent study has proved that mean arterial pressure has independent effects on the blood velocity in middle cerebral artery and internal carotid artery [31]. Therefore, the blood pressure may play an intermediary role in the relevance between the APA therapy and CVHP.

Interestingly, for participants who only received lifestyle interventions in our study, the kinematic indices such as *V*_max_ and *V*_min_ as well as CVHP score at week 2 were a little lower than those at the baseline, and the dynamic indices such as Rv and CP were a little higher, which revealed that the cerebrovascular hemodynamic function was not much improved in the lifestyle intervention alone group. This outcome is consistent with some studies, which similarly found that the advice on lifestyle changes did not affect cerebral blood flow velocity in patients with carotid arteriosclerosis [32]. Nevertheless, some other studies have demonstrated that lifestyle interventions play a positive role in stroke prevention [33, 34]. This inconsistency can be explained in two aspects. First, participants in our study only received a 2-week intervention, and the appearance of a distinctly beneficial effect of lifestyle interventions on the cerebrovascular hemodynamic function may need a long time. Second, the lifestyle interventions for the two groups in our study were only guidance and therefore were greatly influenced by participants' actual adherence to prescribed lifestyle changes [35, 36].

### 4.1. Limitations

Our study has several major limitations. First, the sample size of this study was relatively small. We recruited participants from only one hospital in Hangzhou which is located in East China. As China is a large multiethnic country, the generalizability of our findings is limited. Second, the intervention period was relatively brief, and the effect of long-term APA therapy with lifestyle interventions on cerebrovascular hemodynamic function and stroke prevention needed further verification. Third, only 5 types of auricular points were selected in the study, and the effect of the APA using the other auricular points on cerebrovascular hemodynamic function needed further validation. Fourth, the whole loss to follow-up rate was 18.1%, which may lead to some bias though the dropout rates were almost identical between the two study groups. Fifth, the participants were not blinded due to the current study design, and the knowledge of group assignment may affect their behavior in the study, and therefore, a placebo intervention using seedless patches in the lifestyle intervention alone group would help to blind the participants in a further study. Finally, data on the adverse events, blood pressure, and adherence to lifestyle interventions during the intervention period were not collected, whereas the commonly reported adverse events were usually transient, mild, and tolerable [37].

## 5. Conclusion

In this randomized controlled study, the APA therapy with lifestyle intervention improved the cerebrovascular hemodynamic function and reduced the stroke risk. Therefore, the study demonstrated that the auricular point acupressure can assist the prevention of stroke among subjects who are at high risk of stroke.

## Figures and Tables

**Table 1:** Characteristics of participants at baseline.

Characteristics	APA with lifestyle intervention (*n* = 43)	Lifestyle intervention (*n* = 43)
Gender		
Men, *n* (%)	23 (53.5)	23 (53.5)
Women, *n* (%)	20 (46.5)	20 (46.5)
Age, years	60.56 ± 13.70	60.4 ± 13.10
Lifestyle behavior		
Current smoking, *n* (%)	2 (4.7)	1 (2.3)
Current alcohol intake, *n* (%)	8 (18.6)	6 (14.0)
Weekly physical activity, *n* (%)	38 (88.4)	39 (90.7)
SBP, mmHg	130.12 ± 15.23	135.05 ± 14.31
DBP, mmHg	74.19 ± 9.17	76.06 ± 11.24
Waist, cm	84.95 ± 9.11	88.25 ± 7.11
BMI, kg/m^2^	24.70 ± 3.04	25.59 ± 3.46
FPG, mmol/L	5.22 ± 0.88	5.57 ± 1.47
TC, mmol/L	4.89 ± 1.02	4.96 ± 0.86
TG, mmol/L	1.98 ± 1.69	1.69 ± 0.90
LDL-C, mmol/L	3.04 ± 0.88	3.07 ± 0.66
HDL-C, mmol/L	1.28 ± 0.31	1.33 ± 0.34
Kinematic index		
Left *Q*_mean,_ ml/s	8.23 ± 1.16	8.25 ± 1.05
Right *Q*_mean,_ ml/s	7.85 ± 0.96	7.82 ± 1.08
Left *V*_mean_, cm/s	15.33 ± 2.42	15.25 ± 2.27
Right *V*_mean,_ cm/s	14.38 ± 1.85	14.30 ± 2.22
Left *V*_max,_ cm/s	34.50 ± 5.75	35.23 ± 4.09
Right *V*_max,_ cm/s	33.95 ± 5.27	34.41 ± 4.89
Left *V*_min,_ cm/s	8.05 ± 1.85	7.86 ± 1.89
Right *V*_min,_ cm/s	7.66 ± 1.49	7.57 ± 1.73
Dynamic index		
Left WV, m/s	18.29 ± 6.48	17.79 ± 7.52
Right WV, m/s	18.13 ± 8.02	18.59 ± 9.31
Left Zcv, kPa·km/s	19.21 ± 6.80	18.67 ± 7.90
Right Zcv, kPa·km/s	19.04 ± 8.42	19.52 ± 9.78
Left Rv, kPa·km/s	91.41 ± 16.30	91.48 ± 15.33
Right Rv, kPa·km/s	96.57 ± 14.13	96.93 ± 15.53
Left DR, kPa·km/s	57.98 ± 18.19	59.18 ± 21.93
Right DR, kPa·km/s	62.38 ± 17.44	63.31 ± 20.71
Left CP, kPa	5.10 ± 1.91	4.96 ± 2.31
Right CP, kPa	4.95 ± 1.71	4.83 ± 2.18
Left DP, kPa	4.55 ± 1.43	4.61 ± 1.86
Right DP, kPa	4.68 ± 1.25	4.73 ± 1.64
CVHP score	70.30 ± 10.58	72.51 ± 12.14

Continuous variables are expressed as mean ± SD. APA: auricular point acupressure. SBP: systolic blood pressure. DBP: diastolic blood pressure. BMI: body mass index. FPG: fasting plasma glucose. TC: total cholesterol. TG: triglyceride. LDL-C: low-density lipoprotein cholesterol. HDL-C: high-density lipoprotein cholesterol. *Q*_mean_: mean quantity of carotid blood flow. *V*_mean_: mean velocity of carotid blood flow. *V*_max_: maximal velocity of carotid blood flow. *V*_min_: minimal velocity of carotid blood flow. WV: pulse wave velocity. Zcv: characteristic impendence of vessels. Rv: peripheral resistance of vessels. DR: dynamic resistance. CP: capillary pressure. DP: differential pressure. CVHP: cerebrovascular hemodynamic parameters. SD: standard deviation.

**Table 2:** The kinematic indices at week 2 between two groups.

Kinematic index	Week 2		*P* value	Adjusted *P* value
	APA with lifestyle intervention	Lifestyle intervention		
Left *Q*_mean_, ml/s	8.83 ± 1.04	8.50 ± 1.11	0.075	0.093
Right *Q*_mean_, ml/s	8.34 ± 1.20	7.78 ± 1.07	0.010	0.016⁣^*∗*^
Left *V*_mean_, cm/s	17.30 ± 2.53	15.32 ± 2.23	<0.001	<0.001⁣^*∗*^
Right *V*_mean_, cm/s	15.98 ± 2.65	13.79 ± 2.20	<0.001	<0.001⁣^*∗*^
Left *V*_max_, cm/s	36.77 ± 4.78	34.64 ± 4.22	0.010	0.016⁣^*∗*^
Right *V*_max_, cm/s	35.9 ± 5.68	34.00 ± 4.55	0.045	0.068
Left *V*_min_, cm/s	8.58 ± 1.85	7.59 ± 1.79	0.002	0.005⁣^*∗*^
Right *V*_min_, cm/s	8.16 ± 1.86	7.06 ± 1.70	<0.001	<0.001⁣^*∗*^

Continuous variables are expressed as mean ± SD. APA: auricular point acupressure. *Q*_mean_: mean quantity of carotid blood flow. *V*_mean_: mean velocity of carotid blood flow*. V*_max_: maximal velocity of carotid blood flow *V*_min_: minimal velocity of carotid blood flow. SD: standard deviation. ⁣^*∗*^Statistically significant using the Benjamini–Hochberg procedure.

**Table 3:** The dynamic indices at week 2 between two groups.

Dynamic index	Week 2		*P* value	Adjusted *P* value
	APA with lifestyle intervention	Lifestyle intervention		
Left WV, m/s	15.76 ± 5.25	18.54 ± 5.12	0.006	0.011⁣^*∗*^
Right WV, m/s	16.48 ± 6.80	18.27 ± 6.47	0.220	0.243
Left Zcv, kPa·km/s	16.55 ± 5.51	19.47 ± 5.37	0.006	0.011⁣^*∗*^
Right Zcv, kPa·km/s	17.31 ± 7.14	19.18 ± 6.79	0.220	0.243
Left Rv, kPa·km/s	77.68 ± 13.39	93.25 ± 17.40	<0.001	<0.001⁣^*∗*^
Right Rv, kPa·km/s	83.69 ± 16.11	103.05 ± 18.88	<0.001	<0.001⁣^*∗*^
Left DR, kPa·km/s	44.76 ± 13.96	59.06 ± 20.96	<0.001	<0.001⁣^*∗*^
Right DR, kPa·km/s	48.27 ± 17.06	66.45 ± 23.71	<0.001	<0.001⁣^*∗*^
Left CP, kPa	5.80 ± 1.81	5.43 ± 2.19	0.426	0.426
Right CP, kPa	5.62 ± 2.14	5.19 ± 2.28	0.385	0.404
Left DP, kPa	3.76 ± 1.17	4.38 ± 1.69	0.052	0.073
Right DP, kPa	3.90 ± 1.64	4.59 ± 1.80	0.068	0.089

Continuous variables are expressed as mean ± SD. APA: auricular point acupressure. WV: pulse wave velocity. Zcv: characteristic impendence of vessels. Rv: peripheral resistance of vessels. DR: dynamic resistance. CP: capillary pressure. DP: differential pressure. SD: standard deviation. ⁣^*∗*^Statistically significant using the Benjamini–Hochberg procedure.

**Table 4:** The CVHP score at week 2 between two groups.

Variable	Week 2		*P* value	Adjusted *P* value
	APA with lifestyle intervention	Lifestyle intervention		
CVHP score	85.88 ± 8.89	69.41 ± 13.39	<0.001	<0.001⁣^*∗*^

Continuous variables are expressed as mean ± SD. APA: auricular point acupressure. CVHP: cerebrovascular hemodynamic parameters. SD: standard deviation. ⁣^*∗*^Statistically significant using the Benjamini–Hochberg procedure.

## Data Availability

The data supporting the current study are available from the corresponding author upon request.
